# Antimicrobial resistance and genetic relatedness of *Salmonella* serotypes isolated from food, asymptomatic carriers, and clinical cases in Shiyan, China

**DOI:** 10.1371/journal.pone.0301388

**Published:** 2024-05-09

**Authors:** Jun Lv, Lingjun Geng, Wenlin Ye, Shide Gong, Juan Wu, Tingting Ju, Lin Li, Lanfang Liu, Yonghong Zhang

**Affiliations:** 1 Hubei Key Laboratory of Embryonic Stem Cell Research, Taihe Hospital, School of Basic Medicine, Hubei University of Medicine, Shiyan, China; 2 Health Science Center, Yangtze University, Jingzhou, China; 3 Shiyan Center for Disease Control and Prevention, Shiyan, China; University of Illinois Urbana-Champaign College of Veterinary Medicine, UNITED STATES

## Abstract

*Salmonella* is a primary cause of foodborne diseases globally. Despite food contamination and clinical infections garnering substantial attention and research, asymptomatic *Salmonella* carriers, potential sources of infection, have been comparatively overlooked. In this study, we conducted a comparative analysis of serotype distribution, antimicrobial resistance phenotypes, and genetic profiles of archived *Salmonella* strains isolated from food (26), asymptomatic carriers (41), and clinical cases (47) in Shiyan City, China. Among the 114 *Salmonella* strains identified, representing 31 serotypes and 34 Sequence Types (STs), the most prevalent serovars included Typhimurium, Derby, Enteritidis, Thompson, and London, with the most predominant STs being ST11, ST40, ST26, ST34, and ST155. Antimicrobial resistance testing revealed that all strains were only sensitive to meropenem, with 74.6% showing antimicrobial resistance (AMR) and 53.5% demonstrating multidrug resistance (MDR). Strains resistant to five and six classes of antibiotics were the most common. Pearson’s chi-square test showed no statistically significant difference in the occurrence of AMR (*p* = 0.105) or MDR (*p* = 0.326) among *Salmonella* isolates from the three sources. Our findings underscore associations and diversities among *Salmonella* strains isolated from food, asymptomatic carriers, and clinical patients, emphasizing the need for increased vigilance towards asymptomatic *Salmonella* carriers by authorities.

## Introduction

*Salmonella*, a member of the Enterobacteriaceae family, stands as a significant pathogenic bacterium that affects human and animal health. Defined by the Kauffmann-White scheme, *Salmonella* encompasses more than 2,600 serovars distinguished by three surface antigens: lipopolysaccharide, flagella, and capsular polysaccharide [1]. Globally, an estimated 115 million people, predominantly in developing nations, suffer from *Salmonella* infections annually, resulting in approximately 370,000 deaths [2]. The escalating antimicrobial resistance (AMR) of *Salmonella* poses a pressing threat to both clinical treatment and animal husbandry [3], with the prevalence of multidrug-resistant (MDR) *Salmonella* emerging as a significant concern worldwide [4].

Consumption of contaminated food stands out as the primary route of *Salmonella* infections, followed by person-to-person and animal contact transmission pathways.

The manifestation of symptomatic disease or asymptomatic persistence subsequent to *Salmonella* infection depends on various factors, including the infecting serovars, genetic background, and host immunity. Approximately 3–5% of patients transition into chronic carriers, characterized by prolonged fecal shedding despite the absence of overt signs of infection [5].

Symptomatic diseases disproportionately affect vulnerable populations, such as infants, the elderly, immunocompromised individuals, and those with underlying medical conditions. Asymptomatic patients are individuals who harbor the pathogen without exhibiting clinical symptoms [6,7]. Asymptomatic individuals are often challenging to detect unless they undergo proactive medical testing. Conversely, asymptomatic carriers can manifest symptoms and maintain their ability to transmit the disease. Therefore, the prevalence of carriers represents an urgent public health concern [8].

Several reports have highlighted the circulation of *Salmonella* spp. among animals, humans, and the environment [9]. Typing and tracing *Salmonella* subsequent to isolation and identification are crucial for epidemiological investigations, ensuring food safety, safeguarding public health, and optimizing livestock production [10]. Traditional serotyping and multilocus sequence typing (MLST) are the most commonly employed methods for phenotyping and genotyping, respectively [10].

To date, while some studies have linked foodborne *Salmonella* strains to clinical cases, the understanding of the source and impact of asymptomatic carriers remains limited. In this study, our aim was to investigate serotypes distribution, antimicrobial resistance patterns, and MLST profiles of *Salmonella* isolated from food, asymptomatic carriers, and clinical cases in Shiyan, China. By comparing and analyzing the differences and potential correlations among *Salmonella* isolates from diverse sources, we elucidate the current status of transmission and drug resistance of *Salmonella* in Shiyan. Our findings underscore the imperative for developing more precise and effective strategies to mitigate human *Salmonella* infections.

## Materials and methods

### Study design

This was a cross-sectional study. Archived *Salmonella* strains isolated from food, asymptomatic carriers, and clinical patients between 2018 and 2020 in Shiyan, China were collected and subjected to analysis to explore the associations and diversities in serotype distribution, antimicrobial resistance patterns, and gene profiles.

### Ethical approval

The *Salmonella* strains examined in this article were archived isolates, and all clinical data were anonymized and unlinked. The research content did not entail the use of personal information or involve human blood, tissues, germ cells, embryos, reproductive cloning, chimerism, heritable gene manipulation, or similar activities. In accordance with national ethical review standards for life science and medical research, ethical approval and individual informed consent were deemed unnecessary and exempted.

### Serotyping of *Salmonella* isolates

In our cross-sectional study, we examined 114 previously archived *Salmonella* isolates. Of these, 26 strains were obtained from livestock meat, aquatic products, and ready-to-eat food by foodborne disease inspectors at the Shiyan Center for Disease Control and Prevention. The remaining 88 strains were isolated by the clinical laboratory at Taihe Hospital. Among these, 41 strains were identified from anal swab samples of asymptomatic individuals, while 47 strains were identified from fecal samples of clinical patients. All isolates underwent identification using the VITEK-2 Compact system (bioMerieux Inc., France) and PCR [11,12]. Subsequently, classical serotyping of *Salmonella* isolates was conducted using slide agglutination with O, H, and Vi antigen-specific sera (Diagnostic antisera kit-60 vials, Statens Serum Institute, Denmark) in accordance with the White-Kauffmann-Le Minor scheme [13].

### Antimicrobial susceptibility testing

All *Salmonella* isolates underwent susceptibility testing using Kirby-Bauer disk diffusion method, following the guidelines outlined by the Clinical and Laboratory Standards Institute (CLSI) guidelines [14]. Seventeen commercial antibiotic discs (Liofilchem, Ita) utilized, representing the following 12 classes: (i) aminoglycosides: amikacin (AK, 30 μg), gentamicin (GEN, 10 μg), kanamycin (KAN, 30 μg), streptomycin (STR, 10 μg); (ii) β-lactam combination agents: amoxicillin/clavulanic acid (AMC, 20/10μg); (iii) carbapenems: meropenem (MEM, 10 μg); (iv) cephalosporins: cefazolin (CZO, 30 μg), cefotaxime (CTX, 30 μg); (v) folate pathway inhibitors: trimethoprim/sulphamethoxazole (SXT 25 μg); (vi) monobactams: aztreonam (ATM, 30 μg); (vii) macrolides: azithromycin (AZM, 15 μg); (viii) nitrofurans: nitrofurantoin (NIT, 300 μg); (ix) penicillins: ampicillin (AMP, 10 μg); (x) phenicols: chloramphenicol (CHL, 30 μg); (xi) quinolones: ciprofloxacin (CIP, 5 μg), nalidixic acid (NAL, 30 μg); (xii) tetracyclines: tetracycline (TET, 30 μg).

The pure cultured *Salmonella* colonies were suspended in sterile phosphate-buffered saline to create a 0.5 McFarland inoculum. Subsequently, bacterial suspensions were evenly spread onto Mueller–Hinton agar (Oxoid Ltd., England) using a sterile cotton swab. Following the placement of antibiotic discs on the agar, the plates were allowed to stand for 5 minutes before being incubated at 36°C in ambient air for 18 hours Meanwhile, *E*. *coli* ATCC 25922 was used as a quality-control strain. The diameters of the inhibition zones were measured in millimeters (mm) using a caliper. Interpretation of susceptibility or resistance to each antibiotic adhered to CLSI standards. Multidrug resistance (MDR) was characterized by resistance to at least one drug in each of three or more classes of antimicrobial agents [15].

### Heatmap drawing

A heatmap was generated using TBtools [16] with default parameters to visualize variations in antimicrobial resistances among *Salmonella* isolates from different sources.

### MLST analysis

The total genomic DNA of *Salmonella* isolates were extracted from overnight cultures grown at 37°C in LB broth using a TIANamp Bacteria DNA Kit (TIANGEN, DP302, China) following the manufacturer’s instructions. Subsequently, the extracted DNA were quantified and temporarily stored at -20°C before PCR amplification as templates. MLST of *Salmonella* isolates was conducted according to previously established protocols [17]. Seven housekeeping genes (aroC, dnaN, hemD, hisD, purE, sucA, and thrA) were amplified using the primers listed in S1 Table, purified, and sent to Sangon Biotech (Sangon, Shanghai, China) for bidirectional sequencing. The allele sequences were then compared and analyzed using the *Salmonella* enterica MLST database (http://mlst.warwick.ac.uk/mlst/). Strains that share identical allele gene fragments are assigned to a common sequence type (ST). Furthermore, goeBURST analysis was performed to evaluate the genetic evolution among the different isolates (http://www.phyloviz.net/goeburst/).The primer sequences were obtained from the scheme published on the MLST home page (http://enterobase.warwick.ac.uk/species/senterica/allele_st_search) (S1 Table).

### Statistical analysis

Statistical analysis was conducted using SPSS software, version 27. Differences in antimicrobial resistance and multidrug resistance among *Salmonella* isolated from food, asymptomatic carriers and clinical cases were assessed using Pearson’s chi-square test (χ^2^). A significance level of *p* < 0.05 was considered statistically significant.

## Results

### Distribution of serotypes

To gain a comprehensive understanding of the classification of these samples, we conducted an analysis of serotype distribution. Our findings revealed that among the 114 strains of *Salmonella* examined in this study, 31 serotypes were identified through slide agglutination (Table 1). Notably, *Salmonella* serovar Typhimurium (n = 17, 14.9%) emerged as the predominant serotype in Shiyan between 2018 and 2020, followed by Derby (n = 14, 12.3%), Enteritidis (n = 13, 11.4%), Thompson (n = 10, 8.8%), London (n = 9, 7.9%), Agona (n = 7, 6.1%), Goldcoast (n = 5, 4.4%) and Choleraesuis (n = 4, 3.5%). Senftenberg, Kentucky and Stanley each represented 2.7% (n = 3). Two strains were identified for typhoid, paratyphoid A, paratyphoid B, Bareilly, Liverpool, and Rison, respectively. However, only one isolate was recognized for Anatum, Albany, Corvallis, Give, Hadar, Havana, Lagos, Litchfield, Muenchen, Muenster, Montevideo, Newport, Oranienburg, and Wandsworth, respectively.

**Table 1:** Serotype distribution, MLST profiles and MDR characteristics of 114 *Salmonella* isolates.

Serotypes	Source			STs (n)	MDR%
	Food	Asymptomatic carriers	Clinical cases		
Typhimurium	2	6	9	ST34(9), ST19(7), ST36(1)	64.7%(11/17)
Derby	10	1	3	ST40(13), ST71(1)	71.4%(10/14)
Enteritidis	2		11	ST11(13)	92.3%(12/13)
Thompson	1	6	3	ST26(10)	60.0%(6/10)
London		6	3	ST155(9)	88.9%(8/9)
Agona	2	3	2	ST13(7)	14.3%(1/7)
Gold coast	1	3	1	ST358(5)	20.0%(1/5)
Choleraesuis			4	ST68(4)	100.0%(4/4)
Senftenberg	2	1		ST14(3)	0.0%(0/3)
Kentucky		3		ST198(3)	100.0%(3/3)
Stanley		1	2	ST29(3)	33.3%(1/3)
Bareilly		2		ST203(2)	0.0%(0/2)
Liverpool		2		ST1959(2)	0.0%(0/2)
Paratyphi A			2	ST85(2)	0.0%(0/2)
Paratyphi B			2	ST42(2)	0.0%(0/2)
Rissen		1	1	ST469(2)	0.0%(0/2)
Typhi			2	ST2(2)	0.0%(0/2)
Anatum	1			ST64(1)	0.0%(0/1)
Albany	1			ST292(1)	100.0%(1/1)
Corvallis		1		ST1541(1)	0.0%(0/1)
Give	1			ST516(1)	0.0%(0/1)
Hadar	1			ST33(1)	100.0%(1/1)
Havana		1		ST1527(1)	0.0%(0/1)
Lagos	1			ST1889(1)	0.0%(0/1)
Litchfield			1	ST214(1)	0.0%(0/1)
Muenchen		1		ST82(1)	0.0%(0/1)
Muenster	1			ST321(1)	0.0%(0/1)
Newport			1	ST166(1)	0.0%(0/1)
Oranienburg		1		ST23(1)	0.0%(0/1)
Montevideo		1		ST4(1)	100.0%(1/1)
Wandsworth		1		ST1498(1)	100.0%(1/1)
Total	26	41	47	34	53.5%(61/114)

Abbreviations: ST, sequence type; MDR, multidrug-resistance.

Thirteen, fifteen, and eighteen serotypes were identified among *Salmonella* strains isolated from food (n = 26), asymptomatic carriers (n = 41), and clinical cases (n = 47), respectively. Serovars Derby, Typhimurium, Agona, Thompson, and Goldcoast were detected across isolates from these three sources. Serovars Typhi, Paratyphi A, Paratyphi B, Choleraesuis, Litchfield, and Newport were exclusively isolated from clinical cases. Serovars Anatum, Albany, Give, Hadar, Lagos and Muenster were exclusively isolated from the food source. The five most commonly isolated serovars from food and humans were Typhimurium, Derby, Enteritidis, Thompson, and London. However, the top five prevalent serovars causing human infection were Typhimurium, Enteritidis, Thompson, London, and Agona. Typhimurium and Enteritidis were the most common serovars in humans. These data underscore the diversity of *Salmonella* serovars across the three sources.

### Antimicrobial resistance phenotypes

**Fig 1:**
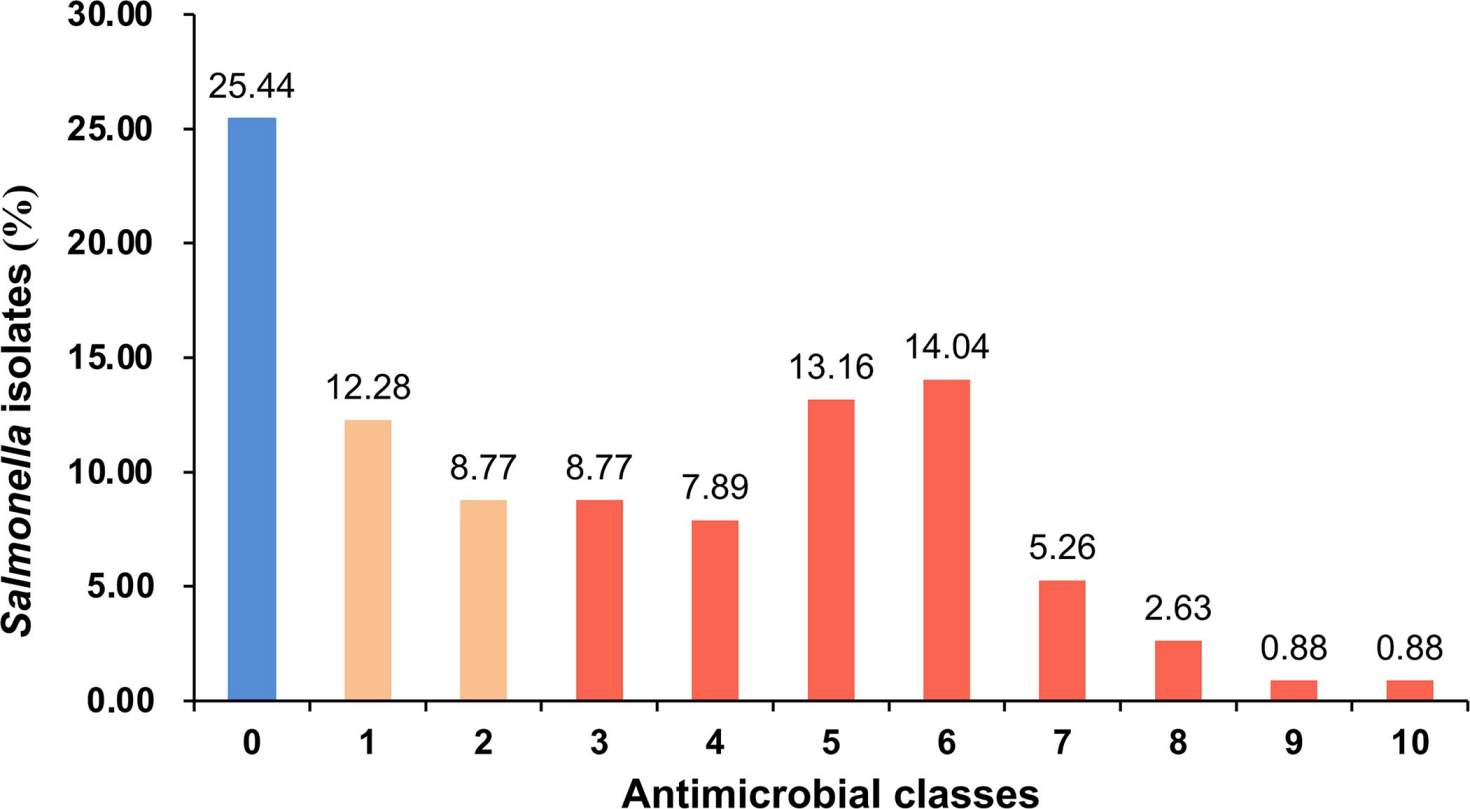
Distribution of antimicrobial resistant (AMR) *Salmonella* strains. As shown, 53.5% of isolates exhibited resistance to three or more antimicrobial classes, defining multidrug resistance (MDR). Different numbers in the x-axis denote the numbers of antimicrobials. Blue, yellow, and orange columns represent susceptibility/intermediate susceptibility, antimicrobial resistance, and multidrug resistance, respectively.

As previously mentioned, monitoring phenotypic antimicrobial resistance is crucial to mitigate the overuse and misuse of antibiotics. We next assessed the susceptibility of *Salmonella* isolates to 17 antibiotics across 12 categories. Of the total, 29 strains (25.44%) exhibited susceptibility to all 12 antimicrobial classes, while 14 (12.28%) and 10 (8.77%) strains displayed resistance to one and two antimicrobial classes, respectively. Notably, the remaining 61 isolates (53.5%) demonstrated resistance to three or more antimicrobial classes, classifying them as MDR strains (Fig 1). Among these, resistance to five and six classes of antimicrobials was notably prevalent (S2 Table). Remarkably, the most resistant strain was *S*. Thompson isolated from clinical cases, exhibiting resistance to 10 classes of antimicrobials.

**Fig 2:**
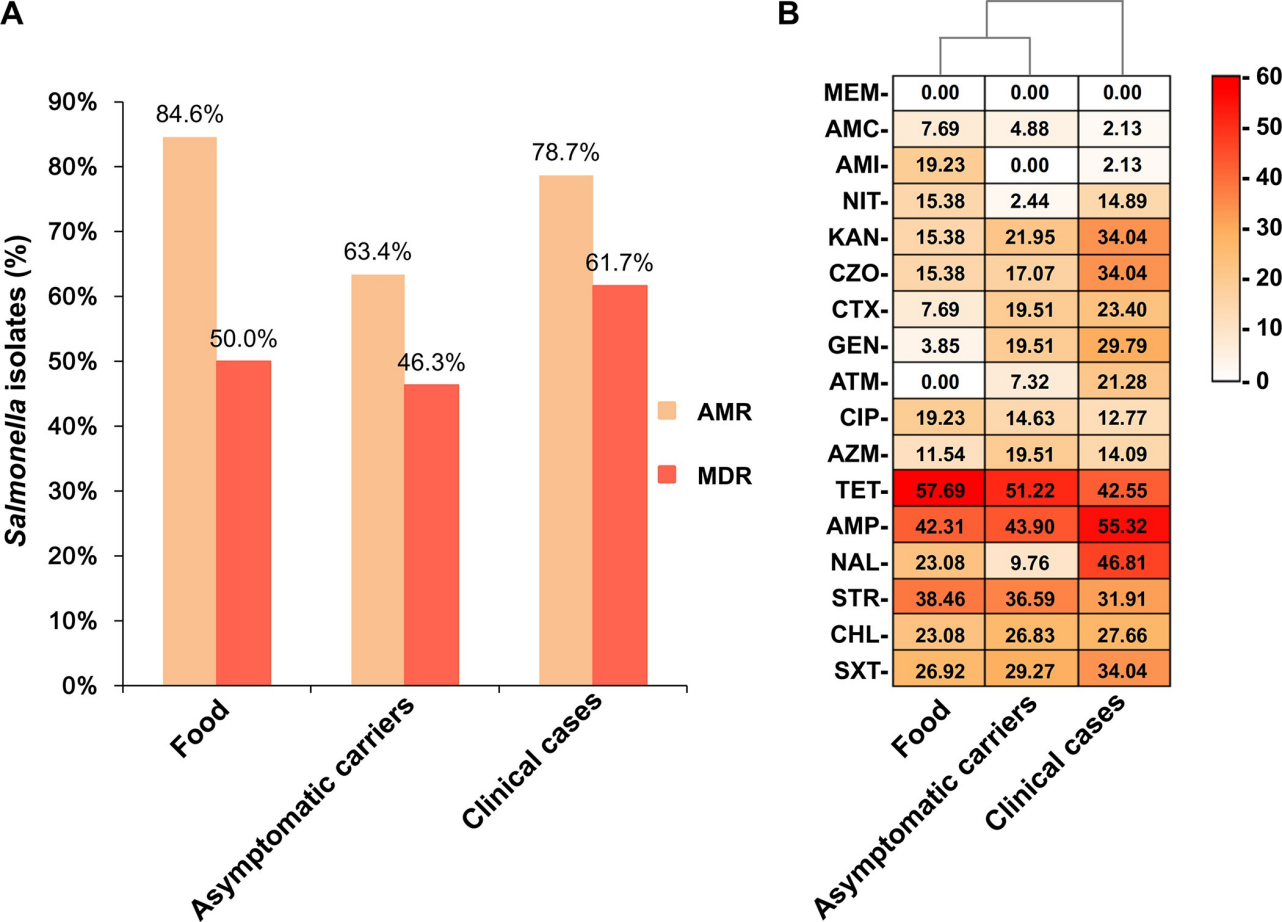
Comparison of antimicrobial resistance in *Salmonella* from different sources. **(A)** The AMR and MDR characteristics of *Salmonella* isolated from food, asymptomatic carriers, and clinical cases are illustrated. Earthy yellow and orange-red columns indicate AMR and MDR, respectively. **(B)** The heatmap depicts the antimicrobial resistance of *Salmonella* strains from various sources to 17 antibiotics. The numbers in cells represent the percentage (%) of antimicrobial resistance isolates. The color bar is located in the upper right corner. Red indicates the resistance rate of *Salmonella* isolates, with darker shades denoting higher resistance rates. Abbreviations: TET: Tetracycline; AMP: Ampicillin; STR: Streptomycin; NAL: Nalidixic acid; SXT: Trimethoprim/sulphamethoxazole; CHL: Chloramphenicol; KAN: Kanamycin; CZO: Cefazolin; GEN: Gentamicin; CTX: Cefotaxime; AZM: Azithromycin; CIP: Ciprofloxacin; ATM:aztreonam; NIT: Nitrofurantoin; AMI:amikacin; AMC: Amoxicillin/clavulanic acid; MEM: Meropenem.

The antimicrobial resistance patterns of *Salmonella* isolated from food, asymptomatic carriers, and clinical cases are depicted in Fig 2A. There was no statistically significant association between strain source and the occurrence of AMR (χ^2^ = 4.501, *p* = 0.105) or MDR (χ^2^ = 2.244, *p* = 0.326), suggesting that the source did not influence the resistance characteristics in this study. The antimicrobial resistance patterns of strains from food and asymptomatic carriers closely resembled each other, whereas those from clinical cases exhibited the most abundant MDR patterns (Fig 2B), indicating that MDR strains tend to be enriched in severe health conditions.

The MDR rates of the top five prevalent *Salmonella* serotypes exceeded the overall MDR rate (53.5%) (Table 1). *S*. Enteritidis (92.3%, 12/13) exhibited the highest MDR rate, followed by *S*. London (88.9%, 8/9), *S*. Derby (71.4%, 10/14), *S*. Typhimurium (64.7%, 11/17), and *S*. Thompson (60.0%, 6/10) (Table 1).

**Fig 3:**
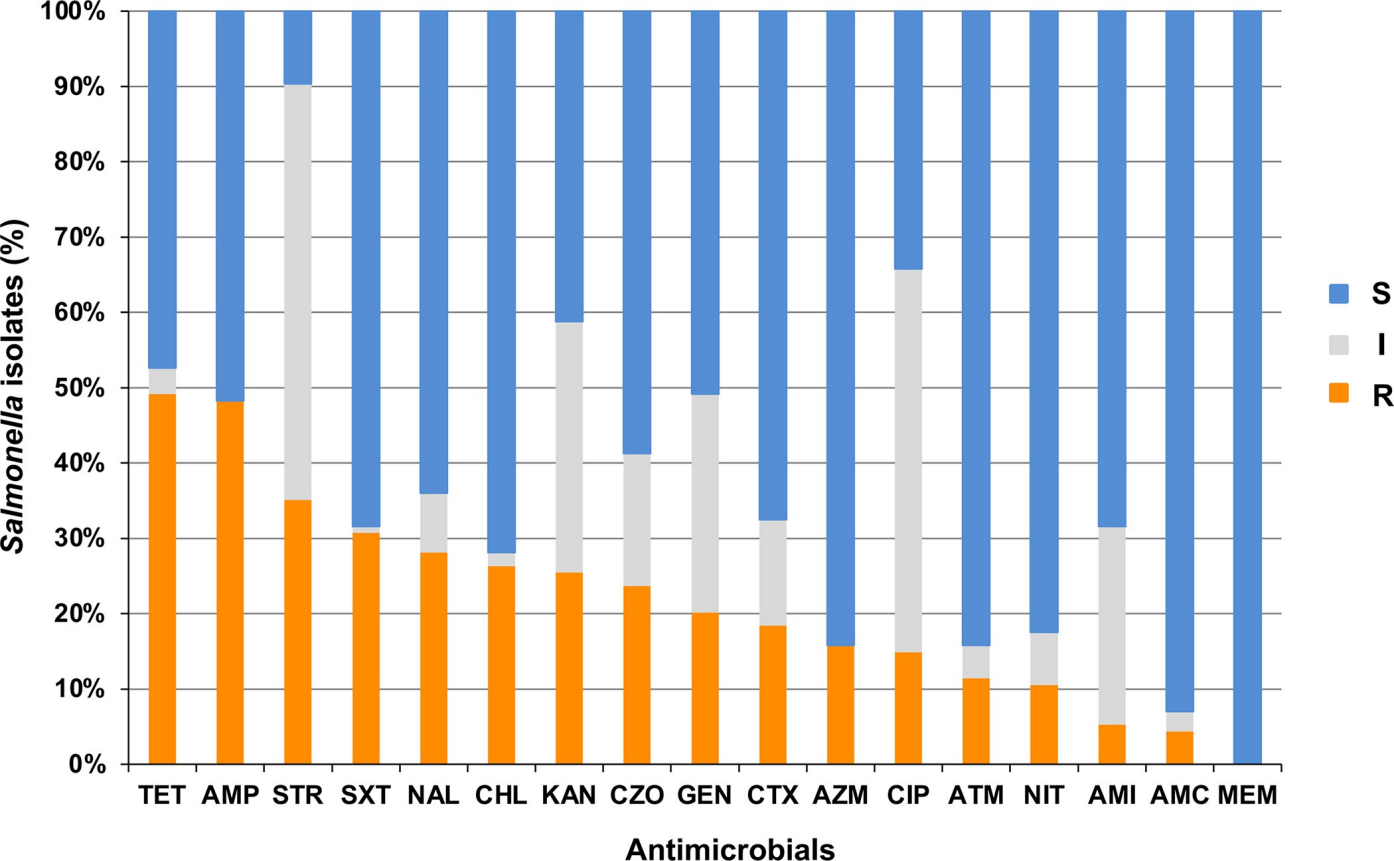
Antimicrobial susceptibility of *Salmonella* isolates to 17 antibiotics. Light blue, gray, and orange columns represent sensitivity, intermediate sensitivity, and resistance, respectively. Column height is proportional to the ratio. Abbreviations: S: Susceptible; I: Intermediate susceptible; R: Resistant. TET: Tetracycline; AMP: Ampicillin; STR: Streptomycin; SXT: Trimethoprim/sulphamethoxazole; NAL: Nalidixic acid; CHL: Chloramphenicol; KAN: Kanamycin; CZO: Cefazolin; GEN: Gentamicin; CTX: Cefotaxime; AZM: Azithromycin; CIP: Ciprofloxacin; ATM:aztreonam; NIT: Nitrofurantoin; AMI:amikacin; AMC: Amoxicillin/clavulanic acid; MEM: Meropenem.

It is noteworthy that we evaluated their responses to 17 antimicrobials, revealing that all 114 isolates in this study demonstrated sensitivity to meropenem, a carbapenem drug utilized in treating symptomatic *Salmonella* infection [18] (Fig 3). *Salmonella* isolates exhibited the highest resistance rate to tetracycline (49.1%, 56/114), followed by ampicillin (48.2%, 55/114), streptomycin (35.1%, 40/114), trimethoprim/sulfamethoxazole (30.7%, 35/114), and nalidixic acid (28.1%, 32/114).

**Fig 4:**
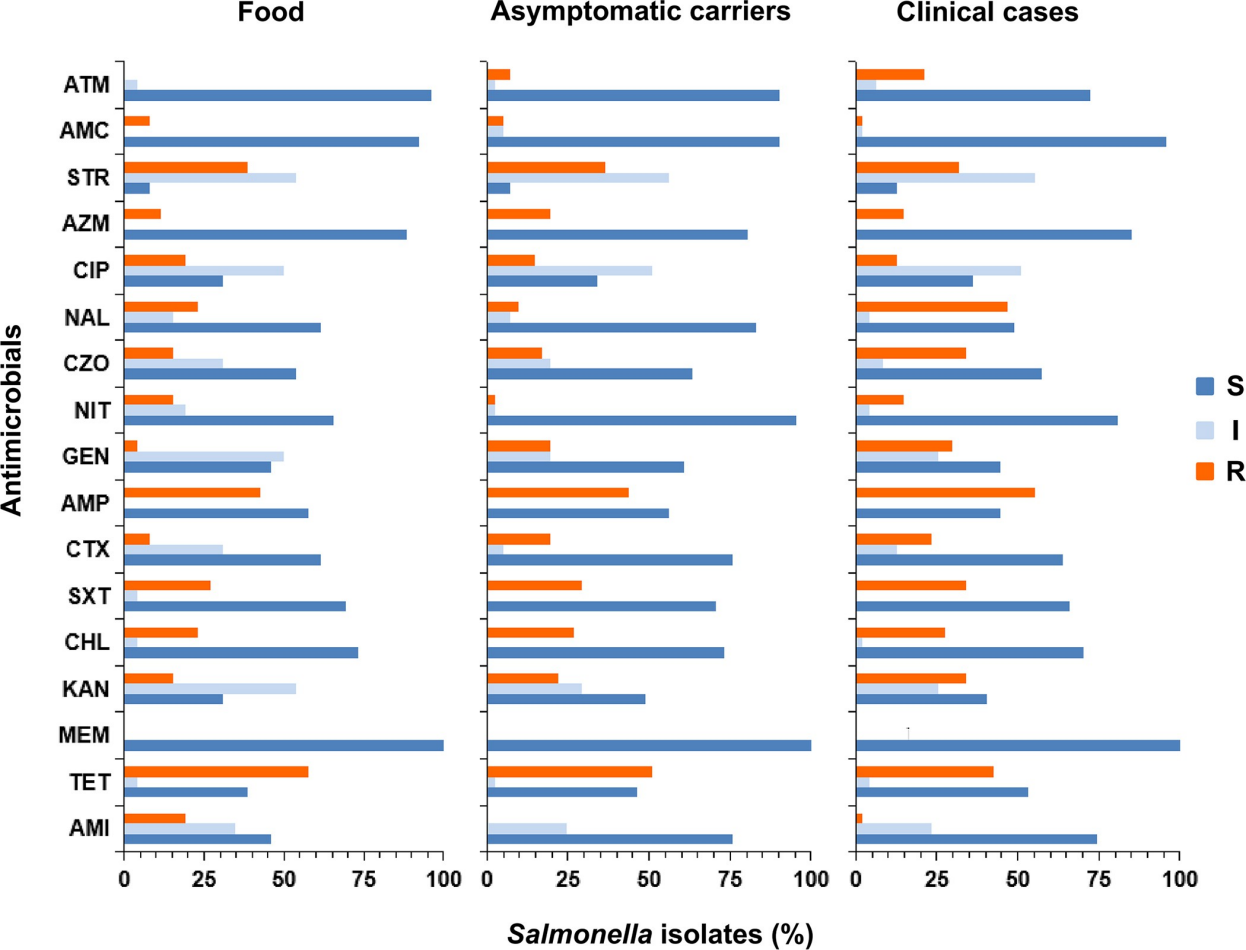
Antimicrobial resistance phenotypes of *Salmonella* isolated from different sources. Blue, gray, and orange columns represent sensitivity, intermediate sensitivity, and resistance, respectively. Column length is proportional to the ratio. Abbreviations: S: Susceptible; I: Intermediate susceptible; R: Resistant. TET: Tetracycline; AMP: Ampicillin; STR: Streptomycin; SXT: Trimethoprim/sulphamethoxazole; NAL: Nalidixic acid; CHL: Chloramphenicol; KAN: Kanamycin; CZO: Cefazolin; GEN: Gentamicin; CTX: Cefotaxime; AZM: Azithromycin; CIP: Ciprofloxacin; ATM: Aztreonam; NIT: Nitrofurantoin; AMI: Amikacin; AMC: Amoxicillin/clavulanic acid; MEM: Meropenem.

Antimicrobial resistance to the aforementioned three antibiotics was also observed in 26 food isolates and 41 asymptomatic carrier isolates. However, among the 47 isolates from clinical cases, ampicillin, tetracycline, and nalidixic acid demonstrated the highest antimicrobial resistance (Fig 4).

### MLST profiles

**Fig 5:**
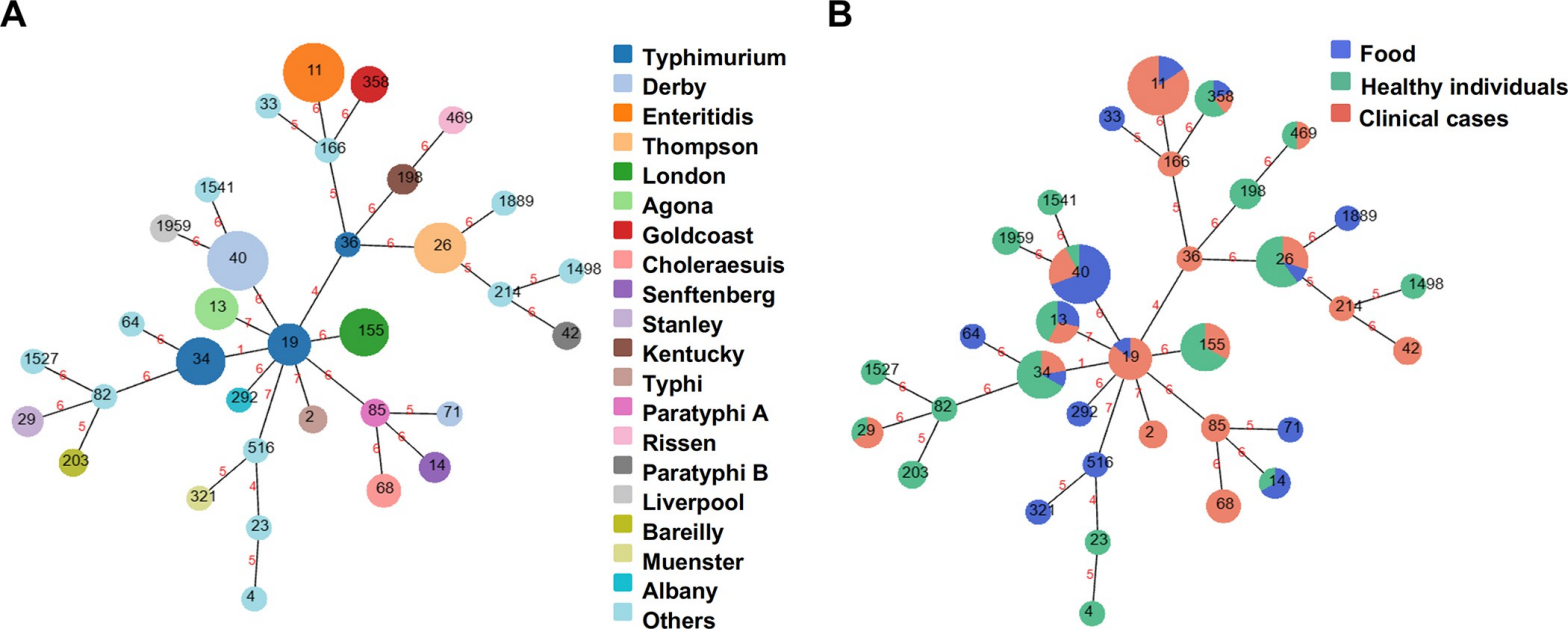
goeBURST diagrams of *Salmonella* isolates based on multilocus sequence typing of seven housekeeping genes presented by (A) serotype and (B) source, respectively. Each circle within the tree represents a single ST (Sequence Type). The size of the circle corresponds to the number of *Salmonella* strains represented. Links between circles are labeled with absolute distance.

MLST, a standard bacterial genotyping method, was utilized to elucidate the genetic relationships among these *Salmonella* strains. From the MLST analysis, the 114 strains were categorized into 34 STs, with ST40, ST11, and ST26 emerging as the most frequent STs, accounting for 11.4% (13/114), 11.4% (13/114), and 8.8% (10/114) of the isolates, respectively (Table 1). It has been observed that *S*. Typhimurium comprises ST19 (7), ST34 (9), and ST36 (1), while *S*. Derby encompasses ST40 (13) and ST71 (1); other serotypes were identified unique STs. A minimum spanning tree, based on the STs obtained for *Salmonella* isolates, was depicted according to serotypes and sources, respectively (Fig 5). It has been shown that sixteen isolates, comprising either ST19 or ST34, were grouped into clonal complex 19, whereas the remaining 98 isolates belonged to 32 distinct clonal complexes. Each of these 32 clonal complexes was associated with only one ST (Fig 5A).

The correlation of STs among strains from different sources is shown in Fig 5B. Five sequence types, namely ST13, ST26, ST34, ST40, and ST358 were identified in samples from in food, asymptomatic carriers, and clinical cases samples. ST11 was associated with both food and clinical cases samples, while ST14 was identified in both food and asymptomatic carriers. ST19, ST29, ST155 and ST469 were found in both clinical cases and asymptomatic carriers. ST33, ST64, ST71, ST292, ST321, ST516 and ST1889 were exclusively recovered from food samples. ST2, ST36, ST42, ST68, ST85, ST166 and ST214 were identified solely in clinical cases samples, while ST4, ST23, ST82, ST198, ST203, ST1498, ST1527, ST1541 and ST1959 were exclusively found in samples from asymptomatic carriers.

## Discussion

*Salmonellosis* ranks as the third leading cause of death among food-transmitted diseases [19,20]. Animal-based foods, including pork, poultry, beef, and seafood, have been extensively implicated as the primary vehicles for the transmission of *Salmonella* spp. to humans. Despite the identification of more than 2600 serovars of S. enterica, human *salmonellosis* is primarily associated with limited serotypes of subspecies enterica. Among these, the top five serovars are Enteritidis, Typhimurium, Infantis, Stanley, and Newport [21]. According to Hendriksen et al., the most commonly isolated serovars exhibit considerable variation across different regions [22].

In this study, the five most commonly isolated serovars from both food and humans were Typhimurium, Derby, Enteritidis, Thompson, and London. However, the top five prevalent serovars causing human infection were Typhimurium, Enteritidis, Thompson, London, and Agona. Typhimurium and Enteritidis emerged as the most common serovars in humans, consistent with reports from numerous countries or regions [23,24]. It is noteworthy that *S*. Typhimurium strains from asymptomatic carriers and clinical cases numbered 9 and 6, respectively, whereas all 11 strains of *S*. Enteritidis were isolated from clinical cases, in accordance with the findings of Xu et al. [25].

The prevalence of serovars Derby, Thompson, and London among the predominant serotypes suggests a potentially high contamination rate in food. A study conducted in Hebei province, China, revealed that these serotypes were the top three *Salmonella* serovars isolated from chicken and pork [26]. While serovar Derby ranked as the most common serotype isolated from food, it only held the sixth position, alongside serovars Gold Coast and Choleraesuis, among the serotypes causing human diseases. Litrup et al. also reported similar observations and suggested that this phenomenon could be attributed to the absence of certain virulence-associated genes in the *Salmonella* pathogenicity island 3 (SPI-3), such as sugR (ATP binding protein) and rhuM (putative cytoplasmic protein) [27].

Compared with the predominant *Salmonella* serotypes in other provinces or cities in China [10,25], the most significant difference observed was the notable increase in serovar Thompson in Shiyan city. *S*. Thompson is seldom pathogenic to poultry; hence, food matrices derived from poultry could serve as vehicles for the transmission of *S*. Thompson to humans through fecal contamination [28]. The sudden surge of serovar Thompson in Shiyan city might indicate that poultry products produced and sold in this area have been significantly contaminated by *Salmonella* Thompson in recent years. The strains of serovar London were isolated from clinical cases (n = 3) and asymptomatic carriers (n = 6) but not from food, suggesting a potential association with insufficient frequency and coverage of food testing.

Significant differences exist in the AMR and MDR characteristics of *Salmonella* isolated from food, humans, and animals [29]. MDR *Salmonella* infections may result in worse outcomes compared to those caused by pan-susceptible strains [30]. In this study, 53.5% (61/114) of *Salmonella* isolates exhibited resistance to at least three classes of antimicrobials. The prevalence of MDR in food isolates (50%) was lower than that reported in Poland (53.8%) but higher than that in Italy (41.6%) [31,32]. The prevalence of MDR in human isolates (asymptomatic carriers, 46.3%, and clinical cases, 61.7%) was lower than that reported in Guizhou (82.9%) and Jiangsu (84.1%) in China but considerably higher than that reported in the European Union (25.4%) [25,33,34]. These variations among different countries may indicate that the abuse or overuse of antibiotics is more prevalent in China compared to European countries.

The antimicrobial resistance of *Salmonella* spp. is a naturally occurring phenomenon, which can be exacerbated by selection pressures stemming from the overuse of antibiotics in medical and livestock breeding practices [35]. In our study, the top five most frequently observed resistant antimicrobials were tetracycline (49.1%), ampicillin (48.2%), streptomycin (35.1%), trimethoprim/sulfamethoxazole (30.7%), and nalidixic acid (28.1%), all of which are commonly used in clinical treatments [36]. Moreover, historically, tetracycline and streptomycin have been widely employed as growth promoters in animal husbandry [37,38]. Meropenem was the only antibiotic observed to be sensitive to all *Salmonella* isolates in our study, consistent with reports from Nantong, China, and Southern Italy [25,39]. Third-generation cephalosporins and fluoroquinolones are the highest priority antibiotics for the treatment of human invasive salmonellosis [29]. Our results showed that among these two classes of antibiotics, *Salmonella* isolates exhibited the highest sensitivity to ciprofloxacin, which was recommended as the adaptive choice for empirical therapy [40,41].

The most resistant isolate was a strain of *S*. Thompson isolated from a clinical case, exhibiting resistance to 10 classes of antimicrobials. To mitigate the worsening *Salmonella* resistance problem, it is imperative to prioritize the rational use of antibiotics by strengthening antimicrobial stewardship in both clinical treatment and animal production [42].

MLST technology has emerged as an ideal tool for global epidemiological investigations owing to its exceptional accuracy, discriminability, and reproducibility [43]. As of February 2023, the PubMLST database (https://pubmlst.org/) had documented 8,818 STs, whereas approximately 2,600 traditional serotypes were reported. MLST analysis not only offers superior discriminatory power compared to serotyping but also enables the prediction of *Salmonella* serotypes [44,45].

The sequence types associated with prevalent serotypes of *Salmonella* have demonstrated geographic and host correlations [46]. In our study, 17 strains of *S*. Typhimurium were categorized into ST19, ST34, and ST36 using MLST, with ST19 and ST34 being the most prevalent, consistent with findings from Shandong and Hangzhou, China [47,48]. However, ST313 exhibits the highest incidence in *S*. Typhimurium infections in sub-Saharan Africa, while ST213 has supplanted ST19 as the predominant genotype of *S*. Typhimurium in Mexico [49,50]. Additionally, we observed that ST19 strains appeared to display higher antimicrobial resistance than ST34 strains, contrary to the findings of Wu et al. [51].

*Salmonella* serovars are categorized into typhoidal and non-typhoidal types. Typhoidal serovars, such as Typhi, Paratyphi, and Sendai, demonstrate high adaptation to humans. Conversely, non-typhoidal serovars possess varying zoonotic potential and can induce human salmonellosis through direct or indirect transmission from animals to humans [52]. Both humans and animals (including swine, cattle, chickens, dogs, cats, ornamental birds, reptiles, amphibians, and rodents) can become asymptomatic carriers following *Salmonella* infection. Serovars Typhimurium, Derby, London, Newport, and Senftenberg commonly cause asymptomatic infections in humans [7,53]. Asymptomatic carriers have the potential to contaminate the environment, water sources, and food by intermittently shedding bacteria in feces over extended periods, thereby posing infection risks to vulnerable populations in the community [54,55]. Research by Parisi et al. suggested that *Salmonella* isolates from asymptomatic individuals were more susceptible to antimicrobials [56]. However, in our study, no statistical difference was observed in the antimicrobial resistance patterns between isolates from food, asymptomatic carriers, and clinical cases.

This study also has limitations. *Salmonella* spp. primarily infects humans through contaminated food. However, due to inadequate frequency and coverage of food sampling, certain *Salmonella* serotypes isolated from human populations may not have been detected in food sources. Due to the relatively small sample size of archived *Salmonella* strains presented, statistical analysis was limited in our study. Instead, we predominantly relied on descriptive methods. In order to draw unbiased conclusions, we recommend conducting a larger sampling across various regions of the country. In the antimicrobial susceptibility test, we selected 17 commonly used antibiotics from 12 classes. If additional antibiotics were included, the antimicrobial resistance profiles of *Salmonella* isolates would likely be more complex. Whole Genome Sequencing (WGS) and Pulsed Field Gel Electrophoresis (PFGE) represent two optimal and efficient approaches for genotyping and exploring genetic relatedness [57]. However, in comparison, the MLST technique incurs lower costs and proved adequate for discriminating between different *Salmonella* strains in this study. With the support of future funding, we anticipate employing WGS to investigate a wider diversity of *Salmonella* strains in subsequent research endeavors.

## Conclusions

This study compared and analyzed the correlation and divergence of serotypes, antimicrobial resistance phenotypes, and genetic profiles of *Salmonella* isolated from food, asymptomatic carriers, and clinical cases. The antimicrobial resistance (AMR) and multidrug resistance (MDR) status of *Salmonella* isolates from asymptomatic carriers was found to be as severe as those from food and clinical cases. Asymptomatic carriers of multidrug-resistant *Salmonella* pose a significant health threat to susceptible populations. Therefore, it is imperative to remind authorities to allocate more attention to asymptomatic *Salmonella* carriers.

## Supporting information

S1 Table Primers used for multilocus sequence typing of *Salmonella* isolates. (DOCX)

S2 Table Antimicrobial resistance patterns of *Salmonella* isolates. (DOCX)
