# Supplementary material for: Antimicrobial resistance and genetic relatedness of Salmonella serotypes isolated from food, asymptomatic carriers, and clinical cases in Shiyan, China
Source: PLoS One. 2024 May 9;19(5):e0301388. doi: 10.1371/journal.pone.0301388 (PMC11081320; doi:10.1371/journal.pone.0301388)
Supplement: S1 Table — (DOCX) [file pone.0301388.s001.docx]

**S1 Table. Primers used for multilocus sequence typing of *Salmonella* isolates**

| Genes | Primers (5`-3`) | | Product (bp) |
| --- | --- | --- | --- |
|  | Amplification | Sequencing |  |
| *aroC* | F: CCTGGCACCTCGCGCTATAC | F: GGCACCAGTATTGGCCTGCT | 826 |
|  | R: CCACACACGGATCGTGGCG | R: CATATGCGCCACAATGTGTTG |  |
| *dnaN* | F: ATGAAATTTACCGTTGAACGTGA | F: CCGATTCTCGGTAACCTGCT | 833 |
|  | R: AATTTCTCATTCGAGAGGATTGC | R: CCATCCACCAGCTTCGAGGT |  |
| *hemD* | F: GAAGCGTTAGTGAGCCGTCTGCG | F: GTGGCCTGGAGTTTTCCACT | 666 |
|  | R: ATCAGCGACCTTAATATCTTGCCA | R: GACCAATAGCCGACAGCGTAG |  |
| *hisD* | F: GAAACGTTCCATTCCGCGCAGAC | F: GTCGGTCTGTATATTCCCGG | 894 |
|  | R: CTGAACGGTCATCCGTTTCTG | R: GGTAATCGCATCCACGAAATC |  |
| *purE* | F: ATGTCTTCCCGCAATAATCC | F: CGCATTATTCCGGCGCGTGT | 510 |
|  | R: TCATAGCGTCCCCCGCGGATC | R: CGCGGATCGGGATTTTCCAG |  |
| *sucA* | F: AGCACCGAAGAGAAACGCTG | F: AGCACCGAAGAGAAACGCTG | 643 |
|  | R: GGTTGTTGATAACGATACGTAC | R: GGTTGTTGATAACGATACGTAC |  |
| *thrA* | F: GTCACGGTGATCGATCCGGT | F: ATCCCGGCCGATCACATGAT | 852 |
|  | R: CACGATATTGATATTAGCCCG | R: CTCCAGCAGCCCCTCTTTCAG |  |
|  | | | |
