# Supplementary material for: Antimicrobial resistance and genetic relatedness of Salmonella serotypes isolated from food, asymptomatic carriers, and clinical cases in Shiyan, China
Source: PLoS One. 2024 May 9;19(5):e0301388. doi: 10.1371/journal.pone.0301388 (PMC11081320; doi:10.1371/journal.pone.0301388)
Supplement: S2 Table — (DOCX) [file pone.0301388.s002.docx]

**S2 Table. Antimicrobial resistance patterns of *Salmonella* isolates**

| **Antimicrobial resistance pattern** | **No. of isolates** | **Proportion**  **(%)** |
| --- | --- | --- |
| None | 29 | 25.44 |
| STR | 3 | 2.63 |
| TET | 3 | 2.63 |
| AMI | 2 | 1.75 |
| AZM | 2 | 1.75 |
| ATM | 1 | 0.88 |
| NAL | 1 | 0.88 |
| NIT | 1 | 0.88 |
| AMP-STR | 1 | 0.88 |
| AMP-TET | 1 | 0.88 |
| KAN-AMI | 1 | 0.88 |
| NAL-ATM | 1 | 0.88 |
| NIT-NAL | 1 | 0.88 |
| TET-CTX | 1 | 0.88 |
| TET-CHL | 1 | 0.88 |
| TET-STR | 1 | 0.88 |
| TET-CHL | 1 | 0.88 |
| AMP-TET-STR | 3 | 2.63 |
| AZM-CIP-NAL | 1 | 0.88 |
| TET-GEN-AMI | 1 | 0.88 |
| TET-SXT-CHL | 1 | 0.88 |
| TET-SXT-GEN | 1 | 0.88 |
| TET-SXT-KAN | 1 | 0.88 |
| TET-STR-CTX | 1 | 0.88 |
| AMP-STR-CZO-NAL | 3 | 2.63 |
| AMP-TET-STR-SXT | 2 | 1.75 |
| AMP-TET-STR-CIP | 1 | 0.88 |
| AMP-TET-STR-KAN | 1 | 0.88 |
| AMP-TET-CHL-SXT | 1 | 0.88 |
| AMP-TET-CHL-CZO-AMC | 1 | 0.88 |
| AMP-TET-CHL-SXT-AZM | 1 | 0.88 |
| AMP-TET-GEN-CHL-SXT | 1 | 0.88 |
| AMP-TET-SXT-NIT-NAL | 1 | 0.88 |
| AMP-TET-STR-SXT-CIP | 1 | 0.88 |
| AMP-TET-KAN-GEN-NAL | 1 | 0.88 |
| AMP-STR-CZO-NAL-NIT | 1 | 0.88 |
| STR-KAN-GEN-SXT-NAL | 1 | 0.88 |
| TET-KAN-SXT-CIP-NAL | 1 | 0.88 |
| AMP-CTX-CZO-KAN-NAL-ATM | 4 | 3.51 |
| AMP-TET-GEN-STR-CHL-SXT | 2 | 1.75 |
| AMP-TET-KAN-CHL-CZO-SXT | 1 | 0.88 |
| AMP-TET-KAN-CHL-GEN-SXT | 1 | 0.88 |
| AMP-TET-STR-CZO-NAL-NIT | 1 | 0.88 |
| TET-STR-KAN-AMI-CIP-NAL | 1 | 0.88 |
| TET-STR-CHL-SXT-NIT-AZM | 1 | 0.88 |
| AMP-TET-STR-GEN-CHL-NAL-AZM | 3 | 2.63 |
| AMP-TET-STR-CHL-CZO-CTX-ATM | 1 | 0.88 |
| AMP-TET-CHL-SXT-CZO-CTX-ATM | 1 | 0.88 |
| AMP-STR-CZO-CTX-NIT-NAL-AZM | 1 | 0.88 |
| AMP-STR-KAN-GEN-CHL-SXT-NAL | 1 | 0.88 |
| AMP-STR-KAN-CHL-CZO-CTX-AZM | 1 | 0.88 |
| AMP-KAN-CZO-CTX-NIT-NAL-ATM | 1 | 0.88 |
| AMP-TET-KAN-GEN-CHL-SXT-CIP-NAL | 2 | 1.75 |
| AMP-TET-KAN-CZO-CTX-CIP-AZM-AMC | 1 | 0.88 |
| AMP-TET-KAN-STR-GEN-CHL-SXT-AZM | 1 | 0.88 |
| AMP-TET-STR-AMI-CHL-SXT-CZO-NAL | 1 | 0.88 |
| AMP-KAN-GEN-CZO-CTX-NIT-NAL-ATM | 1 | 0.88 |
| AMP-TET-KAN-GEN-CHL-SXT-CTX-CIP-NAL | 1 | 0.88 |
| AMP-TET-KAN-SXT-CZO-CTX-AZM-AMC-ATM | 1 | 0.88 |
| AMP-TET-KAN-STR-GEN-CHL-SXT-CZO-AZM | 1 | 0.88 |
| AMP-TET-KAN-GEN-CHL-SXT-NIT-CIP-NAL | 1 | 0.88 |
| AMP-TET-STR-CHL-SXT-CZO-CTX-CIP-AZM | 1 | 0.88 |
| AMP-TET-STR-KAN-GEN-CZO-CTX-CIP-NAL | 1 | 0.88 |
| AMP-TET-STR-CHL-SXT-CZO-CTX-CIP-AZM-AMC | 1 | 0.88 |
| AMP-TET-STR-KAN-GEN-CZO-CTX-CIP-NAL-ATM | 1 | 0.88 |
| AMP-TET-STR-KAN-GEN-CHL-SXT-NIT-CIP-NAL-AZM | 1 | 0.88 |
| AMP-TET-KAN-GEN-CHL-SXT-CZO-CTX-CIP-NAL | 1 | 0.88 |
| AMP-TET-STR-SXT-CZO-CTX-NIT-CIP-NAL-AZM-AMC-ATM | 1 | 0.88 |

Note: Antibiotics highlighted in the same color belong to the same class. Red, blue, and orange denote aminoglycosides, cephalosporins, and quinolones, respectively.
